# Post-operative cardiac arrest induced by co-administration of amiodarone and dexmedetomidine: a case report

**DOI:** 10.1186/s40560-015-0109-0

**Published:** 2015-10-21

**Authors:** Takafumi Ohmori, Nobuhiro Shiota, Akihiro Haramo, Takahiro Masuda, Fumi Maruyama, Kenji Wakabayashi, Yushi U. Adachi, Koichi Nakazawa

**Affiliations:** Department of Intensive Care Medicine, Tokyo Medical and Dental University Medical Hospital, M&D Tower 15th floor, 1-5-45 Yushima, Bunkyo-ku, 1138519 Tokyo Japan

**Keywords:** Amiodarone, Dexmedetomidine, Cardiac arrest

## Abstract

We firstly report a postoperative hemodialysis patient who was co-administered with amiodarone and dexmedetomidine and developed severe bradycardia followed by cardiac arrest. A 79-year-old male patient underwent an amputation of the right lower extremity. The electrocardiogram of the patient showed a complete right bundle branch block with left anterior fascicular block before the anesthesia, and paroxysmal atrial tachycardia over 200 beats/min lasting 15 min was observed during surgery. After admission to the intensive care unit, the intensivist and the consultant cardiologist decided to treat tachycardia using amiodarone. The initial dosing of amiodarone and the maintenance infusion succeeded to decrease the heart rate. Approximately 2 h and a half after the start of dexmedetomidine infusion for sedation, the heart rate gradually declined and severe bradycardia suddenly followed by cardiac arrest was observed. Resuscitation was promptly initiated and the patient regained sinus rhythm without delay. In retrospective analysis, the monitoring record of the electrocardiogram revealed the marked atrioventricular conduction abnormalities. This is the first case report concerning a cardiac arrest induced by amiodarone and dexmedetomidine.

## Background

Dexmedetomidine is one of well-known sedatives, and administration of dexmedetomidine has become a popular regimen in intensive care unit [1–3]. Dexmedetomidine has been considered as a preferable drug among intensivists because of it showing less respiratory and cardiovascular depression [4]. Recently, the application of dexmedetomidine in clinical settings is expanding in Japan and in western countries [1]. However, we previously reported that dexmedetomidine showed a fatal cardiovascular complication including cardiac arrest and reviewed the literature [5]. Now, we firstly present another patient who developed severe bradycardia followed by cardiac arrest induced by the co-administration of amiodarone and dexmedetomidine.

## Case presentation

**Fig. 1:**
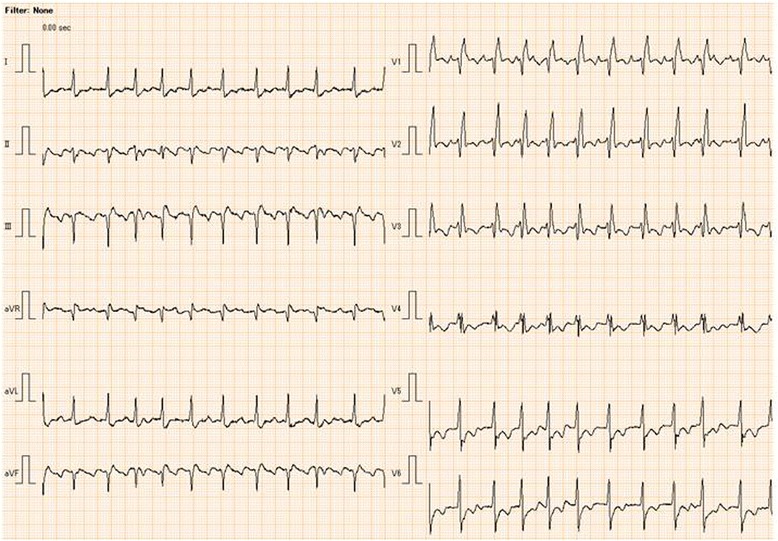
The preoperative electrocardiogram of the patient at admission to the hospital. Complete right bundle branch block with left anterior fascicular block was observed

A 79-year-old male patient was transferred to our hospital for a scheduled amputation surgery of the right lower extremity. He had suffered diabetes and subsequent chronic renal insufficiency. The hemodialysis was introduced 9 years ago in other hospital. The chronic heart failure was pointed out, and the echocardiography demonstrated that the ejection fraction of the left ventricle was only 14 % at the preoperative visit. The history of coronary artery diseases was strongly suspected; however, the detailed information could not be obtained. His electrocardiogram showed sinus rhythm and a sign of complete right bundle branch block with left anterior fascicular block (Fig. 1) [6]. The heart rate was about 130 beats/min, and the QTc interval was 482 ms. The hemodialysis had continued three times a week, and other laboratory data was within normal limits. The patient had been admitted to another hospital, and the sign of arteriosclerosis obliterans was worsening with ischemic change. He was transferred to our hospital for the operation.

The use of local anesthetics on the popliteal sciatic nerve block was planned. Using an ultrasound device, the nerve was identified, and the sufficient amount of analgesia was confirmed after the injection of 20 ml of 0.75 % ropivacaine and 20 ml of 2 % mepivacaine without any complications including paresthesia. Immediately after the beginning of surgery, the anesthesiologist administered propofol at a rate of 2 mg/kg/h; however, the blood pressure of the patient (94/60 mmHg) lowered to 62/42 mmHg, and the infusion was discontinued. During the anesthesia, sudden paroxysmal atrial tachycardia over 200 beats/min was observed. The tachycardia lasted approximately 15 min. The anesthesiologist started to prepare to administer an antiarrhythmic agent (detail was unknown), but the heart rate decreased before the intervention.

**Fig. 2:**
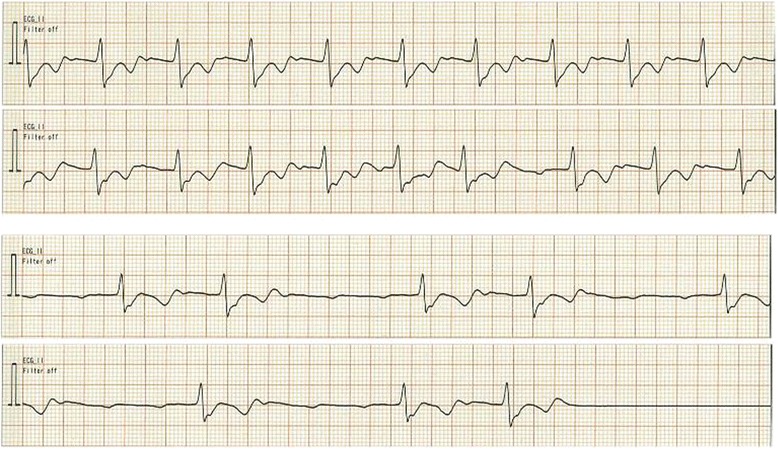
*Upper*: The monitoring record of the electrocardiogram after the administration of amiodarone. Progressive atrioventricular block was observed. *Lower*: The record of the electrocardiogram immediately before the sinus arrest. The complete atrioventricular block with ectopic beat was observed

The patient was admitted to the intensive care unit after the anesthesia for observation. Approximately 2 h later, the heart rate of patient increased to 130–140 beats/min with atrial fibrillation. The intensivist and the consultant cardiologist decided to treat tachycardia with atrial fibrillation using amiodarone. The initial dosing of amiodarone (125 mg/30 min) was followed by fast maintenance dosing (50 mg/h). The heart rate decreased to approximately 100/min during the 2 h after the start of infusion. The infusion rate of amiodarone was reduced to 25 mg/h. After 5 h from the admission to the intensive care unit, infusion of dexmedetomidine was initiated for sedation at a rate of 0.3 μg/kg/h. The heart rate of the patient gradually decreased to 90 beats/min (Fig. 2, upper row). The QTc interval was prolonged to 526 ms. The monitoring electrocardiogram showed sinus rhythm with atrioventricular block.

The dexmedetomidine infusion was continued for 2 h and a half. Then, the heart rate suddenly decreased to 40 beats/min. The complete atrioventricular block along with ectopic rhythm was followed by sinus arrest without escaped beat was occurred (Fig. 2, lower row). Cardiopulmonary resuscitation was promptly initiated. One milligram of epinephrine was administered three times during chest compression and emergency orotracheal intubation. Recovery of spontaneous circulation was observed within 8 min, and the patient regained sinus rhythm and appropriate blood pressure. Mechanical ventilation was started, and spontaneous respiration was observed. Continuous hemodiafiltration was applied for correcting volume abnormalities caused by resuscitation. Immediately after the resuscitation, blood gas analysis showed acidemia (pH 7.05) and hyperlactatemia (13.3 mmol/l); however, both abnormalities disappeared quickly. The infusion of dexmedetomidine was aborted.

Next morning, the patient’s trachea was extubated without any neurological complications. At post-operative day 3, slight liver dysfunction was revealed by laboratory examinations; however, the general condition was improved. The patient was moved to the general ward at post-operative day 6 and discharged to the former hospital at day 10 in good course.

This is the first case report describing a patient presenting cardiac arrest induced by amiodarone and dexmedetomidine. Both drugs are considered as negative chronotropic agents and would suppress the cardiac conduction system. The additive or synergistic interaction on the impulse conduction was strongly suspected for the etiology of cardiac arrest. The preoperative electrocardiogram of the patient showed conduction abnormality, e.g., complete right bundle branch block with left anterior fascicle block, and the conduction dysfunction might be a risk factor for administration of the drugs [5]. We should pay more attention to the possibility of bradycardia; however, the heart rate of the patient showed a consistent tendency of tachycardia before the event.

Amiodarone is one of the most popular antiarrhythmic drugs and widely used [7]. The applicable arrhythmia of amiodarone includes ventricular arrhythmia, e.g., ventricular premature beat, ventricular tachycardia, and supraventricular arrhythmia, e.g., tachycardia with atrial fibrillation. Although amiodarone showed a wide spectrum of antiarrhythmic effect, a variety of complications has been reported on the organs, including the heart, thyroid, liver, eyes, and lungs [8, 9]. The most common complication is bradycardia or conduction disturbance and sympathetic β blocking effect [10]. Kim et al. [10] reviewed that amiodarone-induced bradycardia and atrioventricular block were common adverse effects of amiodarone owing to the calcium channel blocking activity. Amiodarone also significantly prolongs ventricular repolarization, i.e., QTc interval [11], and the effect was consistent with the change in the electrocardiogram of the current patient.

A fatal arrhythmia, ventricular tachycardia, is one of the most recommended conditions to administer amiodarone. We could not find any report describing that amiodarone itself induces cardiac arrest. Tsimogianni et al. [12] reported that administration of itraconazole provoked cardiac arrest in a patient administered with amiodarone. The patient received amiodarone for a treatment of ischemic stroke associated with atrial fibrillation. The administration of itraconazole induced hypotension and subsequent cardiac arrest. The same episode was observed 2 months later, and the antifungal treatment was changed to caspofungin.

Dexmedetomidine decreases heart rate. An incidence of severe bradycardia might be rare [13, 14]; however, we suspect that some of the cases with atrioventricular block induced by the administration of dexmedetomidine were overlooked [5, 15]. Moderate bradycardia with atrioventricular block would be misdiagnosed as a slow sinus rhythm in bipolar electrocardiography monitoring [16]. Usually, intensivists might pay attention to the value of the heart rate, not the pattern of the electrocardiogram. Dexmedetomidine reduces heart rate without a prolongation of the QT interval; thus, QTc interval is shortened [17, 18]. Although the apparent antiarrhythmic effect of dexmedetomidine in human is not known, a possibility of preventing the effect against ventricular tachycardia was demonstrated in animal experiment [19]. Recently, Narisawa et al. [20] demonstrated that dexmedetomidine sedation reduced the incidence of postoperative atrial fibrillation in cardiovascular surgery patients.

## Conclusion

Administration of dexmedetomidine to a patient receiving negative chronotropic drugs should be re-considered, and further attention and intensive monitoring are absolutely required.

## Consent

Written informed consent was obtained from the patient for publication of this case report and any accompanying images. A copy of the written consent is available for review by the Editor-in-Chief of this journal.
